# Complete genome sequence of *Desulfovibrio* sp. GTC20076 isolated from a clinical specimen in Japan

**DOI:** 10.1128/mra.00066-25

**Published:** 2025-04-17

**Authors:** Masahiro Hayashi, Jun Yonetamari, Yoshinori Muto, Kaori Tanaka

**Affiliations:** 1 Institute for Glyco-core Research iGCORE, Gifu University 12785, Gifu, Gifu Prefecture, Japan; 2 Division of Anaerobe Research, Life Science Research Center, Gifu University 201111, Gifu, Gifu Prefecture, Japan; 3 Center for Conservation of Microbial Genetic Resource, Gifu University 12785, Gifu, Gifu Prefecture, Japan; 4 Division of Clinical Laboratory, Gifu University Hospital 476117, Gifu, Gifu Prefecture, Japan; 5 United Graduate School of Drug Discovery and Medical Information Sciences, Gifu University 520344 , Gifu, Gifu Prefecture, Japan; Rochester Institute of Technology, Rochester, New York, USA

**Keywords:** *Desulfovibrio*, complete genome

## Abstract

*Desulfovibrio* is a genus of sulfate-reducing, anaerobic bacteria ubiquitously present in the environment. Herein, we report the complete genome sequence of an isolate of a new *Desulfovibrio* species obtained from a human clinical specimen in Japan. The genome comprised a circular chromosome with a length of 3,213,183 bp.

## ANNOUNCEMENT

*Desulfovibrio* is a genus of mesophilic, gram-negative, anaerobic, rod-shaped bacteria that produce hydrogen sulfide as a terminal by-product of their metabolic activity. These species are commonly found in various environments and can also cause human infections (1, 2). Strain GTC20076 was identified in a human clinical specimen in Aichi, Japan, in 2022. It was isolated from the ascites of an adult patient and cultured on Brucella HK agar with 5% laked sheep blood at 37°C for 48 h under anaerobic conditions (82% N_2_, 10% CO_2_, and 8% H_2_). This research was conducted in accordance with the Declaration of Helsinki. The antibiotic sensitivity of strain GTC20076 was assessed using Dry plate DP-53 (Eiken Chemical Co., LTD, Japan). The strain’s sensitivity to 18 antibiotics was summarized in figshare (10.6084 /m9.figshare.28647680). Identification via matrix-assisted laser desorption/ionization time-of-flight mass spectrometry (MALDI-TOF MS) (Bruker, Germany) confirmed its classification only at the genus level as *Desulfovibrio*.

The genome was sequenced using PromethION 2i (Oxford Nanopore Technologies [ONT], Oxford, UK) for long-read sequencing and the NovaSeq 6000 system (Illumina Inc., USA) for short-read sequencing as previously described (3–5). Genomic DNA was extracted from the pellet using NucleoBond HMW DNA (MACHEREY-NAGEL, Japan). For long-read sequencing, a library was constructed using a Native Barcode Sequencing Kit (SQK-NBD114-24; ONT) without shearing. Sequencing was performed using a FLO-PRO114M flow cell. The raw reads were trimmed and quality filtered using NanoFilt v.2.7.1 (6) with the parameters “-l 1000 -q 10 -headcrop 50.” For short-read sequencing, the DNA Prep (M) Tagmentation kit (Illumina, Inc.) was used for library construction. Subsequently, 2 × 151 bp paired-end sequencing was performed using the NovaSeq 6000 platform. Raw sequencing reads were processed using fastp v.0.20.1 (7) with the parameters “-q 30 -n 20 -t 1 -T 1.” The short reads’ quality was assessed using fastp v.0.20.1 (7). The mean read quality of the long reads was scored using NanoPlot 1.32.1 (7). Short (over 91.9% of bases >Q30 averaged) and long reads (mean read quality of 18.7) were assembled using Unicycler v.0.4.8 (8) with default settings. Assembly circularization and rotation were performed automatically using the Unicycler. The assembly was rotated to start with the *dnaA* gene on the forward strand. Average nucleotide identity (ANI) analysis was conducted using PyANI v.0.2.12 (9).

Table 1 summarizes the genome information. Quality assessment and genome statistics were computed using CheckM (v1.2.2) (10) and seqkit (v2.9.0) (11), respectively. CheckM indicated that the genome was 100% complete with 0% contamination. The DDBJ Fast Annotation and Submission Tool (12) predicted 2,807 coding sequences, nine ribosomal RNAs, 52 transfer RNAs, and one CRISPR sequence.

**TABLE 1:** Information on the complete genome sequence of a *Desulfovibrio* sp. strain isolated from a human clinical specimen in Japan

	Strain name
Parameter	*Desulfovibrio* sp. GTC20076
NovaSeq6000 sequencing^*a*^	
No. of reads	6,261,662
Size (kb)	934,523
Avg coverage (×)	291
DRA accession no.	DRR631455
ONT sequencing^*a*^	
No. of reads	377,632
Size (kb)	3,117,887
Avg read length (bp)	8,256
Avg coverage (×)	970
N50	20,295
DRA accession no.	DRR631456
Assembly	
Assembly N50 (bp)^*c*^	3,213,183
Estimated genome completeness (%)^*d*^	100.0
Estimated genome contamination (%)^*d*^	0.0
Genome structure	1 chromosome
DDBJ/GenBank accession no.	AP039366
Genome size (bp)	3,213,183
GC content (%)	
(chromosome/plasmid name)	57.5
No. of coding sequences^*b*^	2807
Number of rRNAs^*b*^	9
Number of tRNAs^*b*^	52
Number of CRISPRs^*b*^	1

^*a*^ DRA, DDBJ Sequence Read Archive.

^*b*^ DFAST, DDBJ Fast Annotation and Submission Tool.

^*c*^ Determined with seqkit v2.3.0.

^*d*^ Determined with CheckM v1.2.2.

BLASTN analysis (13) of the 16S rRNA gene sequence identified *Desulfovibrio intestinalis* KMS2^T^ as the closest match, with 98.3% identity. However, species identification with the genome sequence using GTDB-tK (v. 2.4.0) was unsuccessful (14). ANI analysis of the complete genome sequences revealed that GTC20076 is most closely related to *Desulfovibrio desulfuricans* (ASM42046v1), with an ANI value of 84.1%, and *Desulfovibrio intestinalis* (ASM1420234v1), with an ANI value of 85.3%. Based on these results, the strain may represent a novel species within the genus *Desulfovibrio*.

## Data Availability

The genome sequences of the *Desulfovibrio* sp. (GTC20076) have been deposited in DDBJ (https://www.ddbj.nig.ac.jp/index.html) under the accession number AP039366. Raw sequence data for GTC20076 have been deposited in the DDBJ/Sequence Read Archive under accession numbers DRR631455 and DRR631456.
